# Cognitive disorders in the elderly persons and their psychometric markers

**DOI:** 10.1192/j.eurpsy.2023.1994

**Published:** 2023-07-19

**Authors:** P. Liubov, Z. Letnikova, E. Daria

**Affiliations:** 1 Department of gerontopsychiatry, Moscow Research Institute of Psychiatry - branch of the V. Serbsky National Medical Research Center for Psychiatry and Narcology, Moscow, Russia, Moscow, Russian Federation

## Abstract

**Introduction:**

Population aging is accompanied by an increase in mental disorders of late age, cognitive impairment and dementia, which makes research on their diagnosis, prevention and therapy particularly relevant.

**Objectives:**

In order to improve the diagnosis of cognitive disorders in elderly patients, a comparative characteristic of the MMSE and MoCA psychometric scales is presented. Due to the lack of differentiation of results in the MoCA - test according to the severity of cognitive disorders, ranking was carried out to determine the scoring levels of cognitive decline.

**Methods:**

On the clinical basis of the scientific department of gerontopsychiatry of the Moscow Research Institute of Psychiatry - branch of the V. Serbsky National Medical Research Center for Psychiatry and Narcology, 46 people over 60 years old were examined. The identified mental disorders were coded under the ICD-10 F00-F09 rubric “Organic, including symptomatic mental disorders”. Cognitive status assessment was carried out by psychometric scales MMSE and MoCA. Psychometric and statistical research methods were used. To compare MoCA and MMSE scores, an equal-percentage alignment method was used

**Results:**

In patients examined by MMSE and MoCA, different point values were determined in assessing cognitive functions. In most observations, the MoCA- test indicators were lower than the MMSE values. The following correspondences of the score values of the scales were revealed: MOCA 23-30 - MMSE 28-30 points; MOCA 22-18 - MMSE 27-25; MOCA 17-12 – MMSE 23-20; MOCA 12-0 – MMSE 19-0.

**Conclusions:**

Such features of the MMSE scale as insufficient sensitivity in assessing memory impairment and differentiation of non-dementia levels of cognitive impairment, regulatory functions of programming and goal-setting, lexical fluency were revealed. The advantages of the MoCA - test were the ability to assess visual-constructive and executive skills, praxis, a more accurate assessment of memory impairments, mobility of mental processes and the ability to switch, logical thinking, the possibility of topical diagnosis of brain damage. The disadvantages of the MoCA - test were the duration of its implementation, the fatigue of patients, the lack of tasks for assessing written speech and motor praxis.

The MoCA - test is a more sensitive method for detecting and differentiating cognitive impairment compared to the MMSE scale, which makes it possible to recommend it for widespread introduction into psychiatric practice. Relevant in further studies are the determination of indicators of moderate cognitive impairment as a threshold value between mild cognitive impairment (MCI) and incipient dementia, clarification of the point values of different levels of dementia.

**Disclosure of Interest:**

None Declared

